# Supplementary figures and images for: Spinal V1 inhibitory interneuron clades differ in birthdate, projections to motoneurons, and heterogeneity
Source: eLife. 2024 Nov 28;13:RP95172. doi: 10.7554/eLife.95172 (PMC11604222; doi:10.7554/eLife.95172)

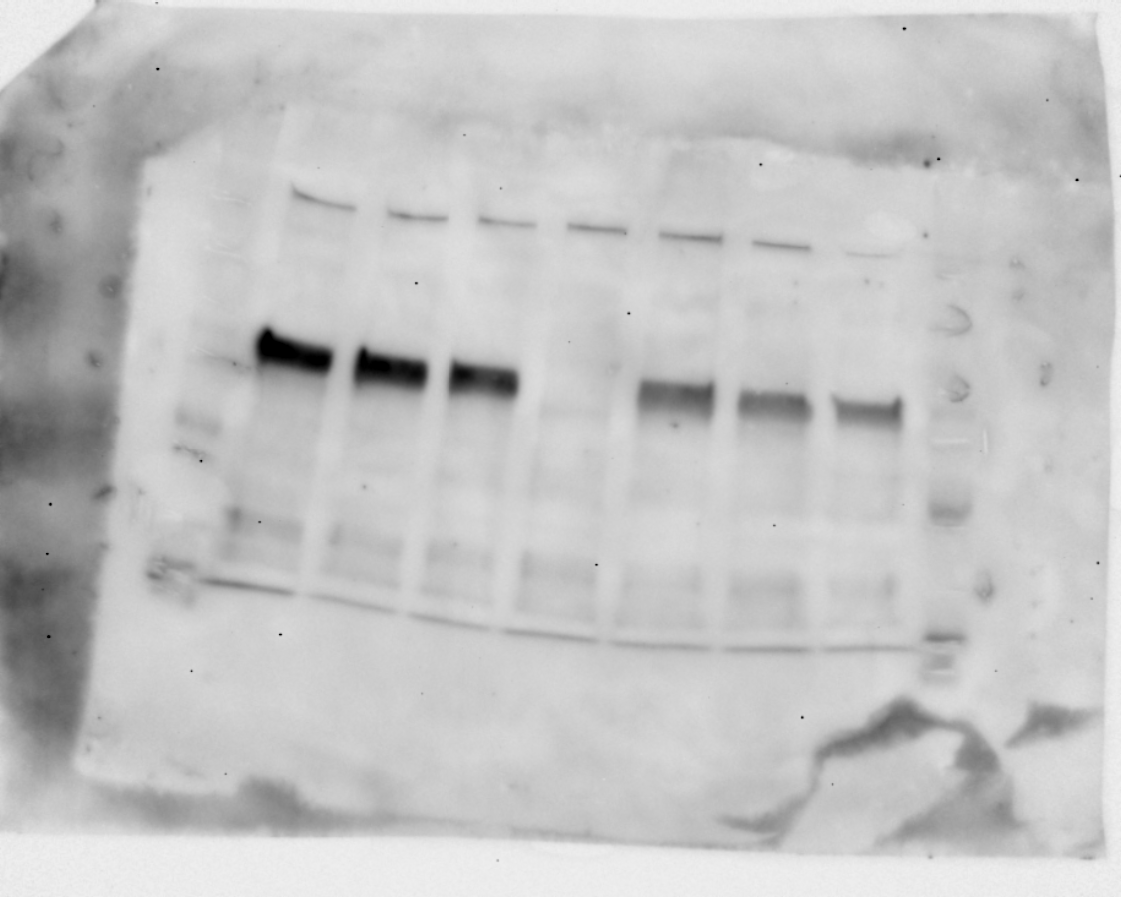

Supplement: Figure 1—figure supplement 1—source data 1. [file elife-95172-fig1-figsupp1-data1.zip › Alvarez 2021-08-06 10h42m35s(Chemiluminescence).tif]

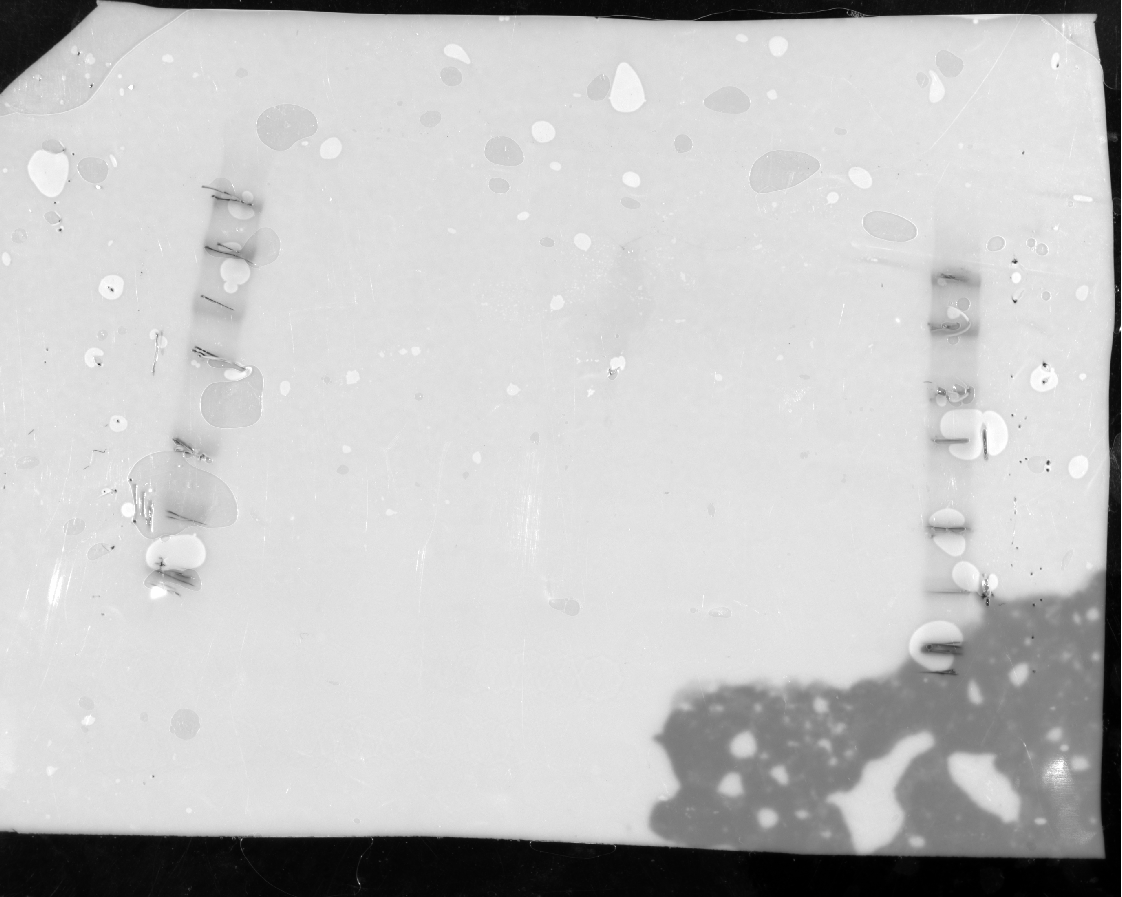

Supplement: Figure 1—figure supplement 1—source data 1. [file elife-95172-fig1-figsupp1-data1.zip › Alvarez 2021-08-06 10h43m46s(Colorimetric).tif]

*Foxp2*(+/+)

*Foxp2*(-/-)

*Foxp2*(+/-)

*Foxp2*(Gt)

Loading  
Control

**kDa**

250

150

100

75

50

37

20

10

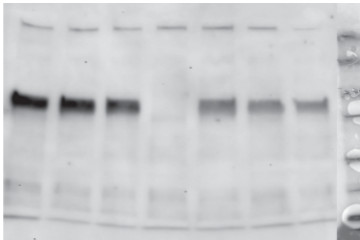

Supplement: Figure 1—figure supplement 1—source data 2. [file elife-95172-fig1-figsupp1-data2.pdf]

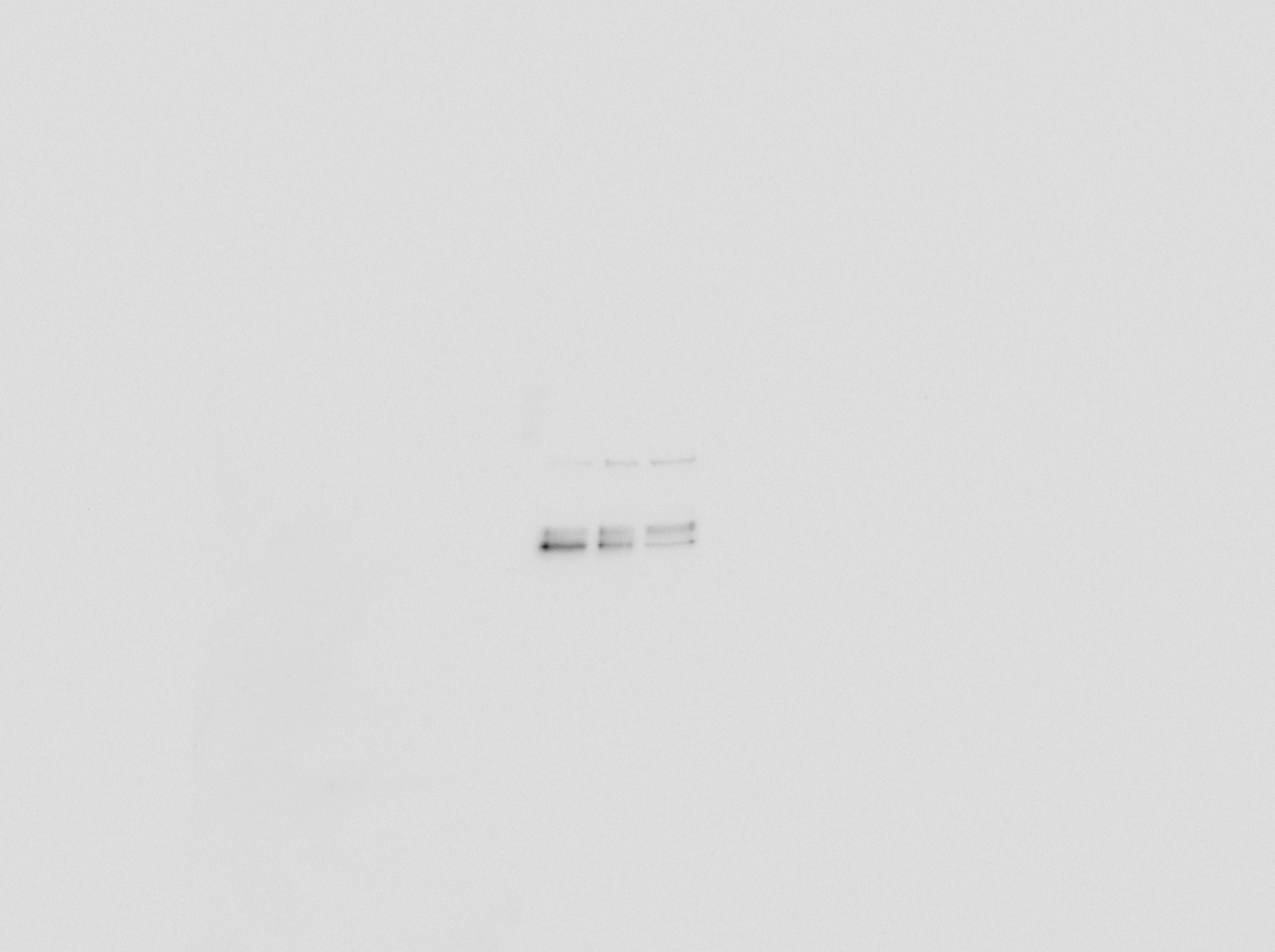

Supplement: Figure 1—figure supplement 2—source data 1. [file elife-95172-fig1-figsupp2-data1.zip › Griffith Maf AB Westren probe 3 Sigma MafB antibody.tif]

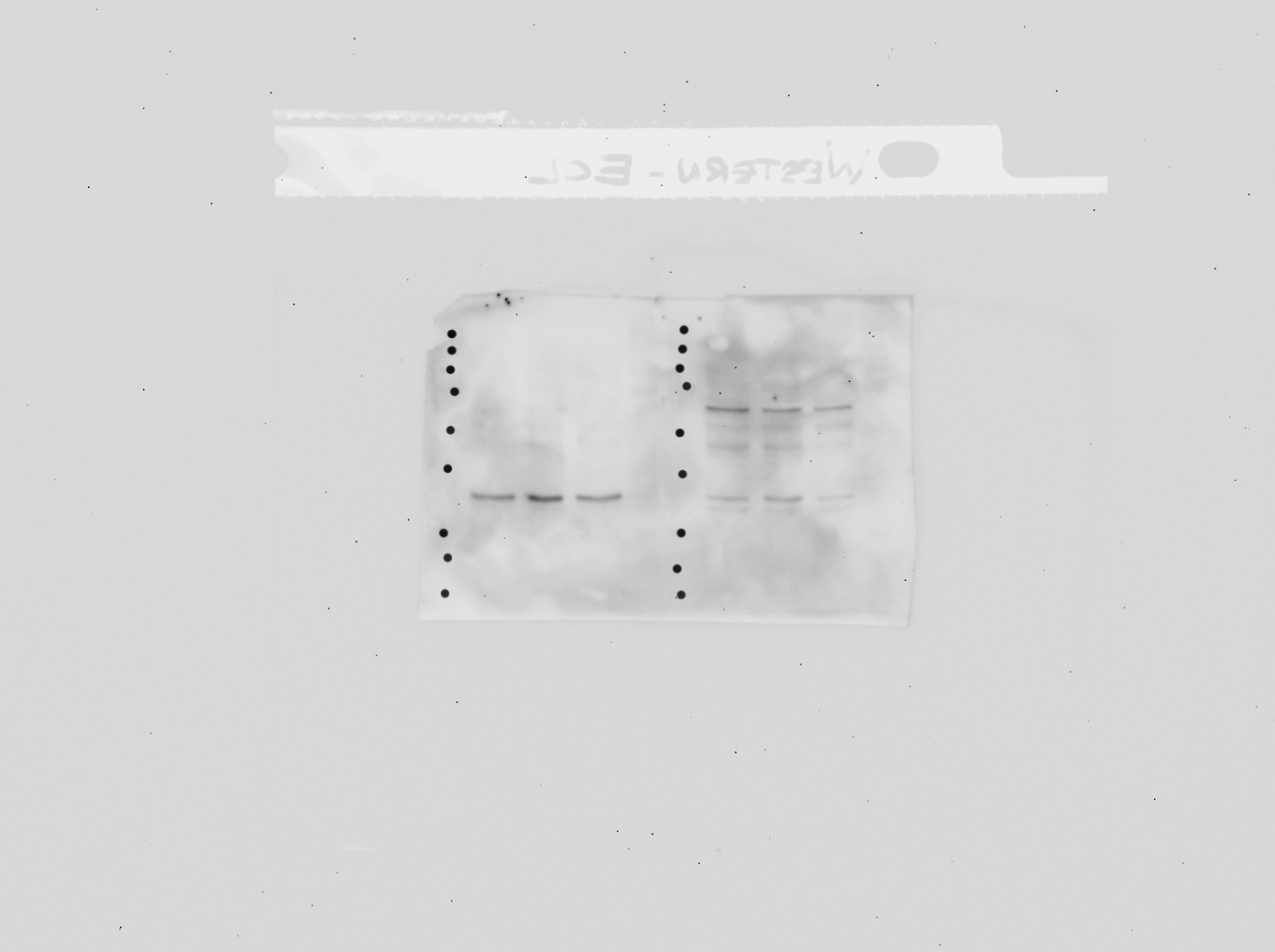

Supplement: Figure 1—figure supplement 2—source data 1. [file elife-95172-fig1-figsupp2-data1.zip › Griffith Novus Maf A replicate (left) and cMaf antibody (right).tif]

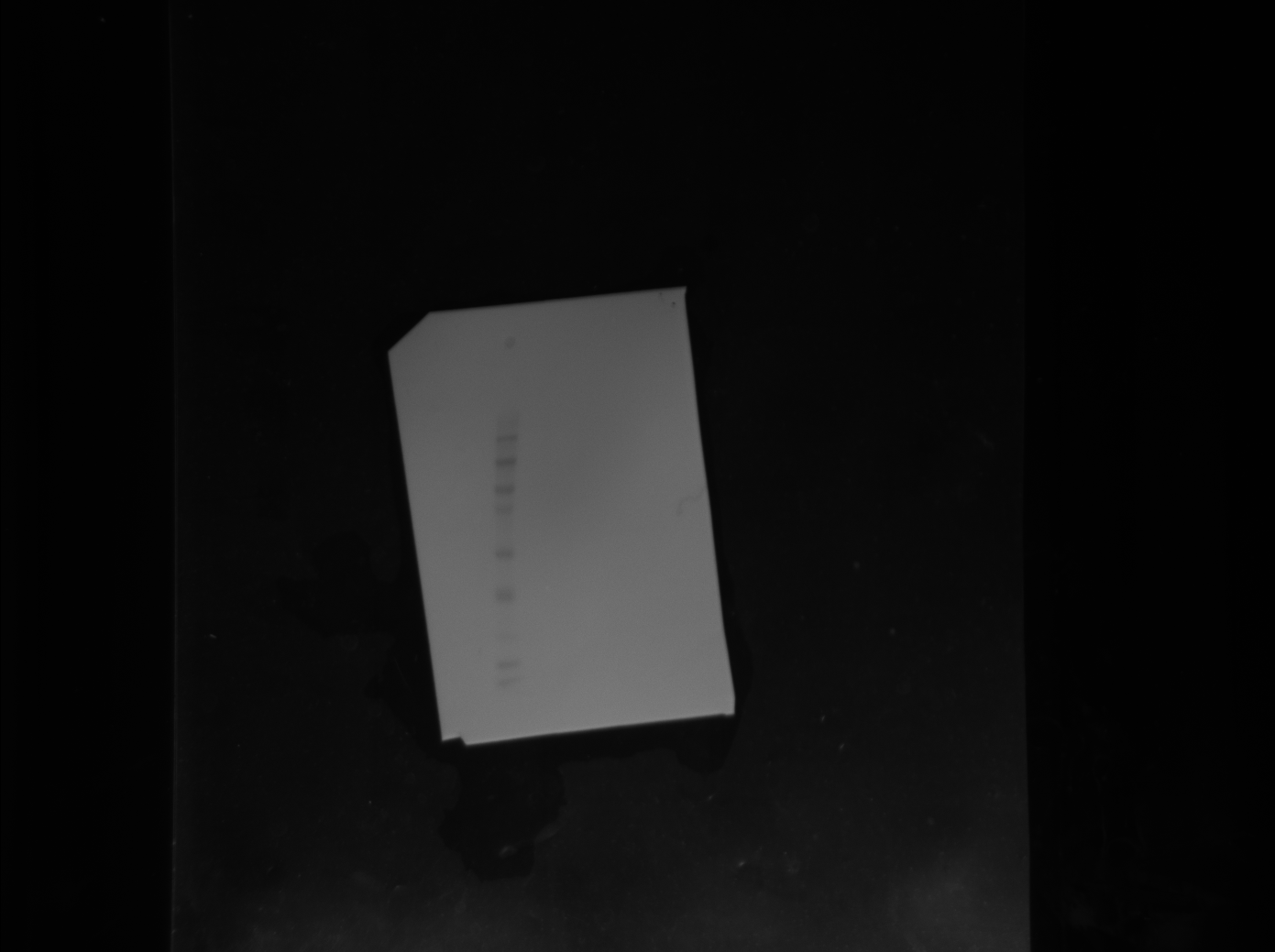

Supplement: Figure 1—figure supplement 2—source data 1. [file elife-95172-fig1-figsupp2-data1.zip › Griffith Maf AB Western colorimetric standards.tif]

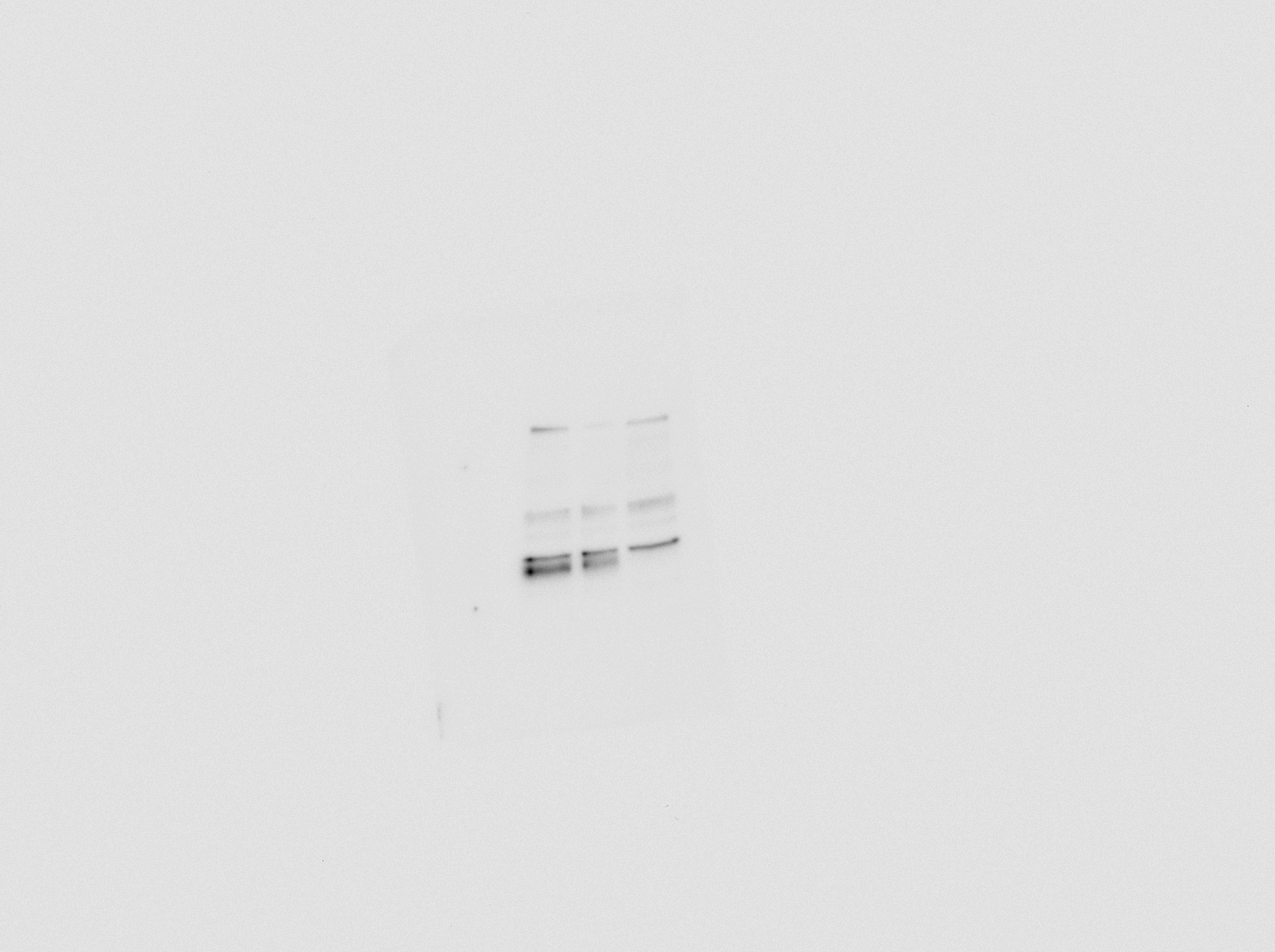

Supplement: Figure 1—figure supplement 2—source data 1. [file elife-95172-fig1-figsupp2-data1.zip › Griffith Maf AB Western probe 1 Novus MafB antibody.tif]

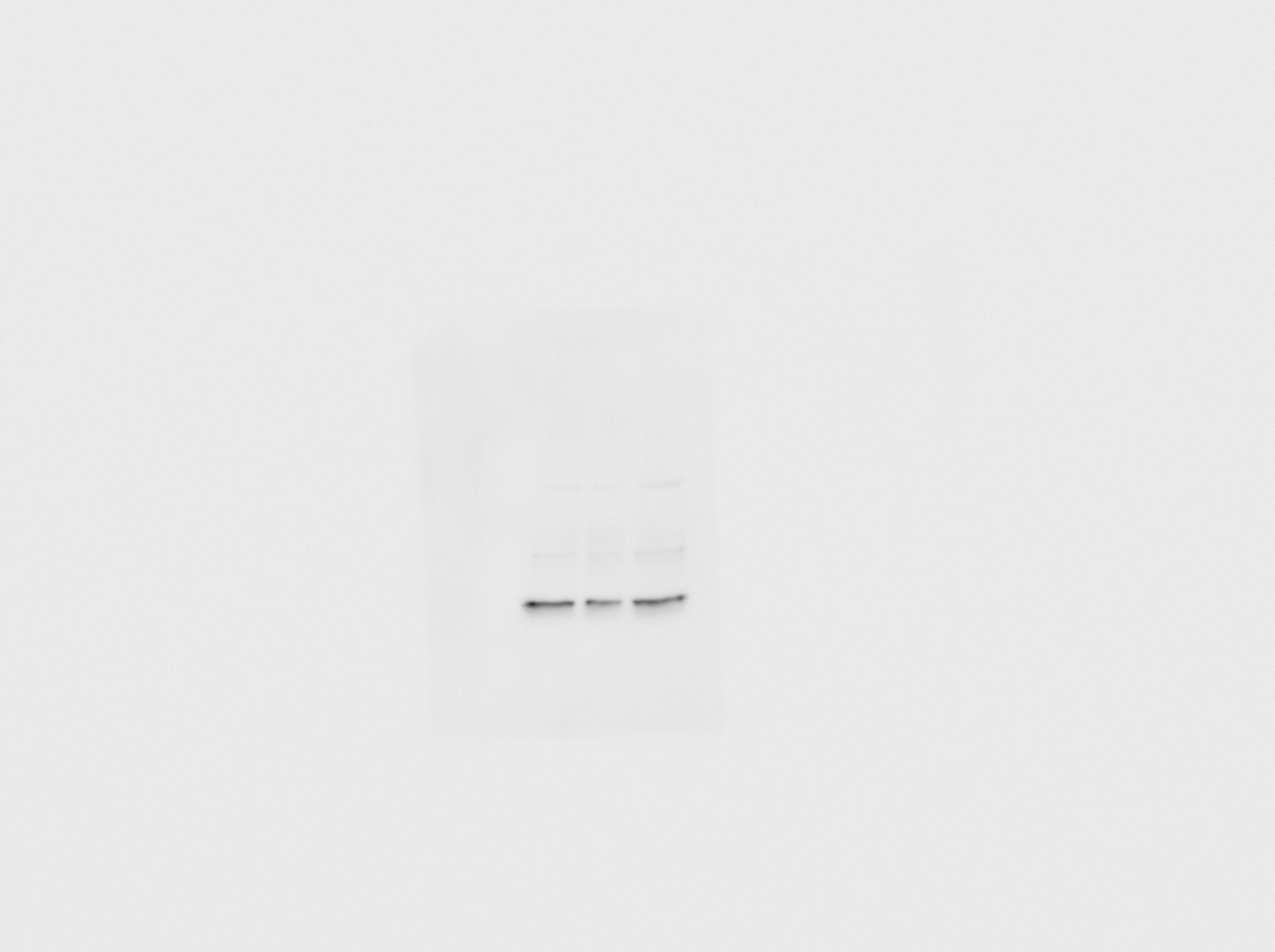

Supplement: Figure 1—figure supplement 2—source data 1. [file elife-95172-fig1-figsupp2-data1.zip › Griffith Maf AB Western probe 2 Novus MafA antibody.tif]

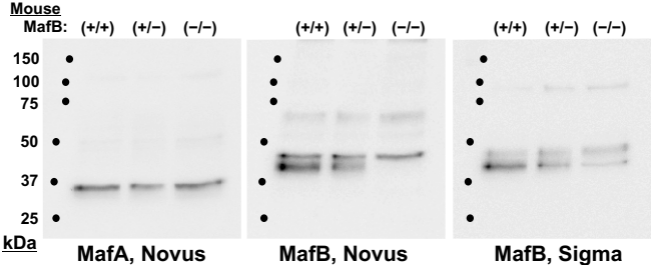

Supplement: Figure 1—figure supplement 2—source data 2. [file elife-95172-fig1-figsupp2-data2.pdf]

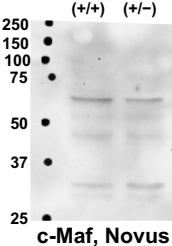

Supplement: Figure 1—figure supplement 2—source data 3. [file elife-95172-fig1-figsupp2-data3.pdf]

Otp::Flpo

---

Wt

KI

Wt

10—

5—

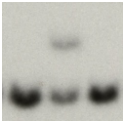

BamH1 Digest;  
5' Probe

Supplement: Figure 7—figure supplement 1—source data 2. [file elife-95172-fig7-figsupp1-data2.pdf]
