# Supplementary material for: Spinal V1 inhibitory interneuron clades differ in birthdate, projections to motoneurons, and heterogeneity
Source: eLife. 2024 Nov 28;13:RP95172. doi: 10.7554/eLife.95172 (PMC11604222; doi:10.7554/eLife.95172)
Supplement: Figure 7—figure supplement 1—source data 1. [file elife-95172-fig7-figsupp1-data1.pdf]

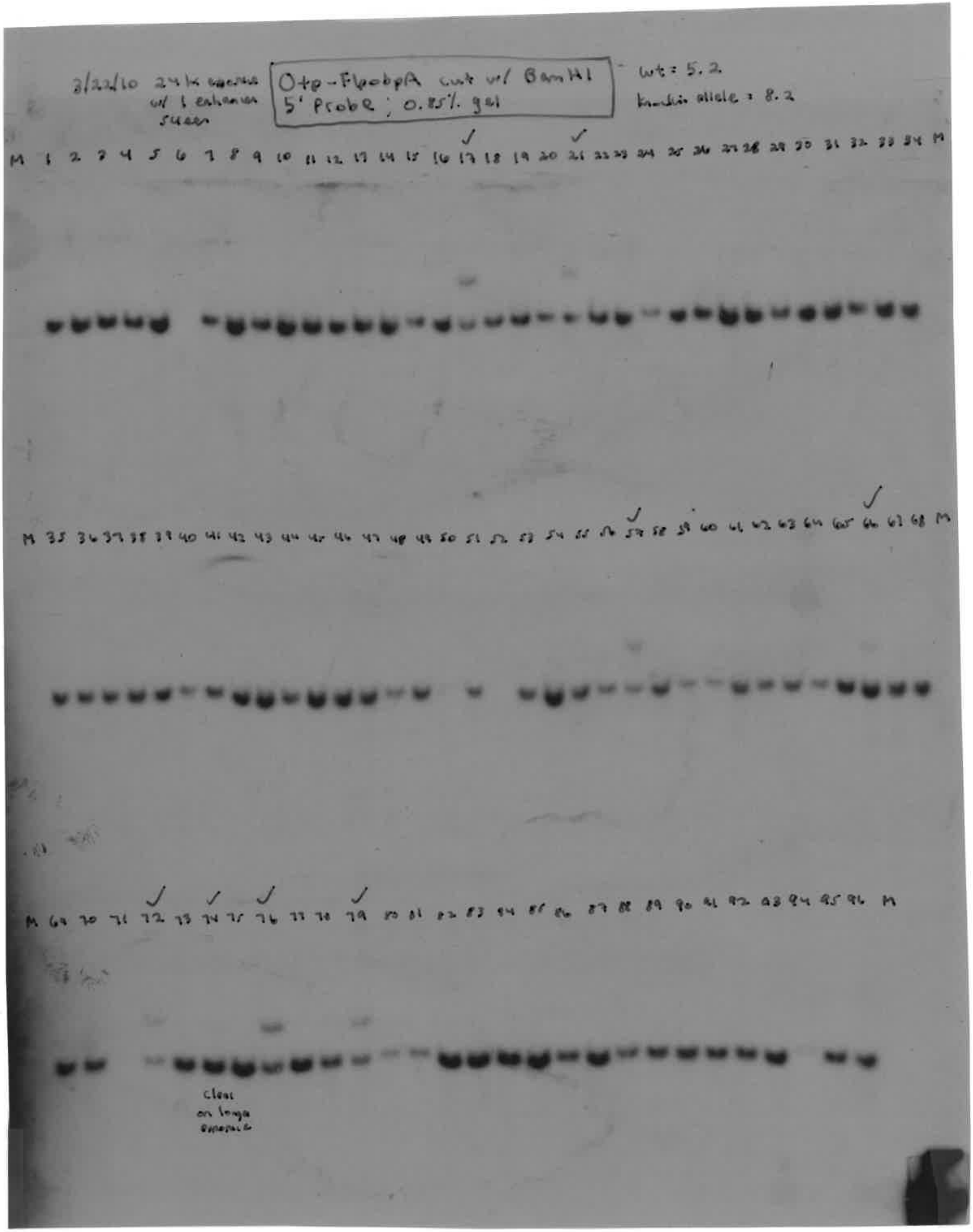
3/22/10 24 hr exposure w/ 1 enhancer screen
Otp-FlpobpA cut w/ BamHI
5' Probe; 0.85% gel
wt = 5.2
knockin allele = 8.2
M 1 2 3 4 5 6 7 8 9 10 11 12 13 14 15 16 17 18 19 20 21 22 23 24 25 26 27 28 29 30 31 32 33 34 M
M 35 36 37 38 39 40 41 42 43 44 45 46 47 48 49 50 51 52 53 54 55 56 57 58 59 60 61 62 63 64 65 66 67 68 M
M 69 70 71 72 73 74 75 76 77 78 79 80 81 82 83 84 85 86 87 88 89 90 91 92 93 94 95 96 M
Clear on longer exposure
