# Supplementary material for: Spinal V1 inhibitory interneuron clades differ in birthdate, projections to motoneurons, and heterogeneity
Source: eLife. 2024 Nov 28;13:RP95172. doi: 10.7554/eLife.95172 (PMC11604222; doi:10.7554/eLife.95172)
Supplement: Supplementary file 1. — Contains all the statistical tables for graphs shown in figures. 1a is related to Figure 4D; 1b is related to Figure 5D; 1c is related to Figure 5E, top graph; 1d is related to Figure 5E, bottom graph; 1e is related to Figure 6C, left graph Otp/Foxp2; 1f is related to Figure 6C, center graph Foxp4/Foxp2; 1g is related to Figure 6C, right graph Otp/Foxp4. [file elife-95172-supp1.docx]

Supplementary File 1

Statistical Tables 1a to 1g

**Spinal V1 inhibitory interneuron clades differ in birthdate, projections to motoneurons, and heterogeneity.**

**Abbreviated title**: Diversity of V1 Spinal Interneurons

**Authors**

Andrew E. Worthy^1,2^, JoAnna T. Anderson^2^, Alicia R. Lane^2^, Laura Gomez-Perez^2^, Anthony A. Wang^1^, Ronald W. Griffith^1,2^, Andre F. Rivard^2^, Jay B. Bikoff^3^, Francisco J. Alvarez^1,2#^

^1^ Department of Physiology, Emory University School of Medicine, Atlanta, GA, USA 30322

^2^ Department of Cell Biology, Emory University School of Medicine, Atlanta, GA, USA 30322

^3^ Department of Developmental Neurobiology, St Jude Children’s’ Research Hospital, Memphis, TN, USA 38105

**Supplementary file 1a;** related to Figure 4D.

| Ordinary one-way ANOVA summary |  |
| --- | --- |
| F | 43.30 |
| P value | <0.0001 |
| P value summary | **** |
| Significant diff. among means (P < 0.05)? | Yes |
| R squared | 0.9319 |

| Bonferroni's multiple comparisons test | Mean Diff. | 95.00% CI of diff. | t (df:19) | Summary | Adjusted P Value |
| --- | --- | --- | --- | --- | --- |
| Th13 vs. L1 | -11.39 | -26.33 to 3.552 | 2.670 | ns | 0.3179 |
| Th13 vs. L2 | -14.72 | -28.70 to -0.7460 | 3.690 | * | 0.0327 |
| Th13 vs. L3 | -27.47 | -41.45 to -13.50 | 6.885 | **** | <0.0001 |
| Th13 vs. L4/5 | -41.50 | -55.48 to -27.52 | 10.40 | **** | <0.0001 |
| Th13 vs. L6 | -42.07 | -55.43 to -28.70 | 11.03 | **** | <0.0001 |
| Th13 vs. S1 | -0.111 | -15.05 to 14.83 | 0.026 | ns | >0.9999 |
| L1 vs. L2 | -3.333 | -17.31 to 10.64 | 0.835 | ns | >0.9999 |
| L1 vs. L3 | -16.08 | -30.06 to -2.107 | 4.031 | * | 0.0150 |
| L1 vs. L4/5 | -30.11 | -44.09 to -16.13 | 7.546 | **** | <0.0001 |
| L1 vs. L6 | -30.68 | -44.04 to -17.31 | 8.041 | **** | <0.0001 |
| L1 vs. S1 | 11.28 | -3.663 to 26.22 | 2.644 | ns | 0.3362 |
| L2 vs. L3 | -12.75 | -25.69 to 0.1895 | 3.451 | ns | 0.0562 |
| L2 vs. L4/5 | -26.78 | -39.72 to -13.84 | 7.249 | **** | <0.0001 |
| L2 vs. L6 | -27.34 | -39.62 to -15.07 | 7.802 | **** | <0.0001 |
| L2 vs. S1 | 14.61 | 0.6349 to 28.59 | 3.662 | * | 0.0348 |
| L3 vs. L4/5 | -14.03 | -26.97 to -1.088 | 3.797 | * | 0.0256 |
| L3 vs. L6 | -14.59 | -26.87 to -2.319 | 4.164 | * | 0.0111 |
| L3 vs. S1 | 27.36 | 13.38 to 41.34 | 6.857 | **** | <0.0001 |
| L4/5 vs. L6 | -0.567 | -12.84 to 11.71 | 0.162 | ns | >0.9999 |
| L4/5 vs. S1 | 41.39 | 27.41 to 55.37 | 10.37 | **** | <0.0001 |
| L6 vs. S1 | 41.96 | 28.59 to 55.32 | 11.00 | **** | <0.0001 |

**Supplementary file1b;** related to Figure 5D.

Nested One-Way ANOVA F_(11, 80)_ = 36.09; p < 0.0001

Variation within animals: SD = 3.018, Variance = 9.108

Inter-animal variation for each motor column/segment: Chi square_(1)_ = 0.5061; p = 0.4768

| Bonferroni's multiple comparisons test | Mean Diff. | 95.00% CI of diff. | t (df:80) | Summary | Adjusted P Value |
| --- | --- | --- | --- | --- | --- |
| Th12/Th13 HMC vs. L1/L2 LMCv | 0.7566 | -2.819 to 4.332 | 0.741 | ns | >0.9999 |
| Th12/Th13 HMC vs. L4/L5 LMCd | -3.142 | -6.515 to 0.2315 | 3.262 | ns | 0.1074 |
| Th12/Th13 HMC vs. L4/L5 LMCv | -5.112 | -8.384 to -1.839 | 5.471 | **** | <0.0001 |
| Th12/Th13 HMC vs. L6 LMCd | 0.8142 | -2.697 to 4.325 | 0.812 | ns | >0.9999 |
| Th12/Th13 HMC vs. Th12/Th13 MMC | 2.695 | -0.8050 to 6.195 | 2.697 | ns | 0.5632 |
| Th12/Th13 HMC vs. L1/L2 MMC | 2.551 | -0.9381 to 6.040 | 2.561 | ns | 0.8136 |
| Th12/Th13 HMC vs. L3/L4 MMC | -0.3096 | -3.682 to 3.062 | 0.321 | ns | >0.9999 |
| Th12/Th13 HMC vs. S1/2 MMC | 2.373 | -0.9170 to 5.663 | 2.526 | ns | 0.8913 |
| Th12/Th13 HMC vs. Th12/Th13 PGC | 7.144 | 3.876 to 10.41 | 7.658 | **** | <0.0001 |
| Th12/Th13 HMC vs. L1/L2 PGC | 7.097 | 3.642 to 10.55 | 7.195 | **** | <0.0001 |
| Th12/Th13 HMC vs. S1/S2 PGC | 5.473 | 2.110 to 8.836 | 5.700 | **** | <0.0001 |
| L1/L2 LMCv vs. L4/L5 LMCd | -3.898 | -7.275 to -0.5210 | 4.043 | ** | 0.0080 |
| L1/L2 LMCv vs. L4/L5 LMCv | -5.868 | -9.145 to -2.592 | 6.273 | **** | <0.0001 |
| L1/L2 LMCv vs. L6 LMCd | 0.05760 | -3.457 to 3.572 | 0.057 | ns | >0.9999 |
| L1/L2 LMCv vs. Th12/Th13 MMC | 1.938 | -1.565 to 5.442 | 1.938 | ns | >0.9999 |
| L1/L2 LMCv vs. L1/L2 MMC | 1.794 | -1.699 to 5.287 | 1.799 | ns | >0.9999 |
| L1/L2 LMCv vs. L3/L4 MMC | -1.066 | -4.442 to 2.310 | 1.106 | ns | >0.9999 |
| L1/L2 LMCv vs. S1/2 MMC | 1.616 | -1.678 to 4.911 | 1.719 | ns | >0.9999 |
| L1/L2 LMCv vs. Th12/Th13 PGC | 6.387 | 3.116 to 9.659 | 6.838 | **** | <0.0001 |
| L1/L2 LMCv vs. L1/L2 PGC | 6.341 | 2.882 to 9.800 | 6.421 | **** | <0.0001 |
| L1/L2 LMCv vs. S1/S2 PGC | 4.716 | 1.349 to 8.084 | 4.906 | *** | 0.0003 |
| L4/L5 LMCd vs. L4/L5 LMCv | -1.970 | -5.024 to 1.084 | 2.259 | ns | >0.9999 |
| L4/L5 LMCd vs. L6 LMCd | 3.956 | 0.6471 to 7.264 | 4.187 | ** | 0.0048 |
| L4/L5 LMCd vs. Th12/Th13 MMC | 5.836 | 2.540 to 9.133 | 6.200 | **** | <0.0001 |
| L4/L5 LMCd vs. L1/L2 MMC | 5.692 | 2.407 to 8.977 | 6.069 | **** | <0.0001 |
| L4/L5 LMCd vs. L3/L4 MMC | 2.832 | -0.3288 to 5.993 | 3.138 | ns | 0.1572 |
| L4/L5 LMCd vs. S1/2 MMC | 5.514 | 2.441 to 8.588 | 6.285 | **** | <0.0001 |
| L4/L5 LMCd vs. Th12/Th13 PGC | 10.29 | 7.236 to 13.33 | 11.82 | **** | <0.0001 |
| L4/L5 LMCd vs. L1/L2 PGC | 10.24 | 6.990 to 13.49 | 11.04 | **** | <0.0001 |
| L4/L5 LMCd vs. S1/S2 PGC | 8.615 | 5.463 to 11.77 | 9.575 | **** | <0.0001 |
| L4/L5 LMCv vs. L6 LMCd | 5.926 | 2.720 to 9.132 | 6.474 | **** | <0.0001 |
| L4/L5 LMCv vs. Th12/Th13 MMC | 7.807 | 4.613 to 11.00 | 8.561 | **** | <0.0001 |
| L4/L5 LMCv vs. L1/L2 MMC | 7.663 | 4.481 to 10.84 | 8.435 | **** | <0.0001 |
| L4/L5 LMCv vs. L3/L4 MMC | 4.802 | 1.749 to 7.855 | 5.509 | **** | <0.0001 |
| L4/L5 LMCv vs. S1/2 MMC | 7.485 | 4.523 to 10.45 | 8.850 | **** | <0.0001 |
| L4/L5 LMCv vs. Th12/Th13 PGC | 12.26 | 9.319 to 15.19 | 14.62 | **** | <0.0001 |
| L4/L5 LMCv vs. L1/L2 PGC | 12.21 | 9.065 to 15.35 | 13.60 | **** | <0.0001 |
| L4/L5 LMCv vs. S1/S2 PGC | 10.58 | 7.542 to 13.63 | 12.18 | **** | <0.0001 |
| L6 LMCd vs. Th12/Th13 MMC | 1.881 | -1.557 to 5.318 | 1.916 | ns | >0.9999 |
| L6 LMCd vs. L1/L2 MMC | 1.737 | -1.690 to 5.163 | 1.775 | ns | >0.9999 |
| L6 LMCd vs. L3/L4 MMC | -1.124 | -4.431 to 2.184 | 1.190 | ns | >0.9999 |
| L6 LMCd vs. S1/2 MMC | 1.559 | -1.665 to 4.783 | 1.694 | ns | >0.9999 |
| L6 LMCd vs. Th12/Th13 PGC | 6.330 | 3.129 to 9.530 | 6.926 | **** | <0.0001 |
| L6 LMCd vs. L1/L2 PGC | 6.283 | 2.891 to 9.675 | 6.488 | **** | <0.0001 |
| L6 LMCd vs. S1/S2 PGC | 4.659 | 1.361 to 7.957 | 4.947 | *** | 0.0003 |
| Th12/Th13 MMC vs. L1/L2 MMC | -0.1440 | -3.559 to 3.271 | 0.148 | ns | >0.9999 |
| Th12/Th13 MMC vs. L3/L4 MMC | -3.004 | -6.300 to 0.2912 | 3.193 | ns | 0.1330 |
| Th12/Th13 MMC vs. S1/2 MMC | -0.3218 | -3.533 to 2.890 | 0.351 | ns | >0.9999 |
| Th12/Th13 MMC vs. Th12/Th13 PGC | 4.449 | 1.261 to 7.637 | 4.887 | *** | 0.0003 |
| Th12/Th13 MMC vs. L1/L2 PGC | 4.403 | 1.022 to 7.783 | 4.562 | ** | 0.0012 |
| Th12/Th13 MMC vs. S1/S2 PGC | 2.778 | -0.5082 to 6.065 | 2.961 | ns | 0.2664 |
| L1/L2 MMC vs. L3/L4 MMC | -2.860 | -6.144 to 0.4236 | 3.051 | ns | 0.2044 |
| L1/L2 MMC vs. S1/2 MMC | -0.1778 | -3.378 to 3.022 | 0.195 | ns | >0.9999 |
| L1/L2 MMC vs. Th12/Th13 PGC | 4.593 | 1.417 to 7.769 | 5.064 | *** | 0.0002 |
| L1/L2 MMC vs. L1/L2 PGC | 4.547 | 1.178 to 7.916 | 4.727 | *** | 0.0006 |
| L1/L2 MMC vs. S1/S2 PGC | 2.922 | -0.3526 to 6.197 | 3.125 | ns | 0.1633 |
| L3/L4 MMC vs. S1/2 MMC | 2.683 | -0.3893 to 5.754 | 3.059 | ns | 0.1997 |
| L3/L4 MMC vs. Th12/Th13 PGC | 7.453 | 4.406 to 10.50 | 8.566 | **** | <0.0001 |
| L3/L4 MMC vs. L1/L2 PGC | 7.407 | 4.159 to 10.65 | 7.987 | **** | <0.0001 |
| L3/L4 MMC vs. S1/S2 PGC | 5.783 | 2.633 to 8.933 | 6.430 | **** | <0.0001 |
| S1/2 MMC vs. Th12/Th13 PGC | 4.771 | 1.814 to 7.727 | 5.652 | **** | <0.0001 |
| S1/2 MMC vs. L1/L2 PGC | 4.724 | 1.562 to 7.887 | 5.232 | **** | <0.0001 |
| S1/2 MMC vs. S1/S2 PGC | 3.100 | 0.03801 to 6.162 | 3.546 | * | 0.0434 |
| Th12/Th13 PGC vs. L1/L2 PGC | -0.04630 | -3.185 to 3.093 | 0.052 | ns | >0.9999 |
| Th12/Th13 PGC vs. S1/S2 PGC | -1.671 | -4.708 to 1.367 | 1.926 | ns | >0.9999 |
| L1/L2 PGC vs. S1/S2 PGC | -1.624 | -4.863 to 1.614 | 1.757 | ns | >0.9999 |

.

**Supplementary file 1c**; related to Figure 5E, top graph.

**Foxp2-V1 vs non-Foxp2-V1 synapses**

Two-Way ANOVA for Synapse Origin and Motor Column/Segment

- Column/Segment: F_(11, 248)_ = 22.52, p < 0.0001
- Foxp2 vs Non-Foxp2: F_(1, 248)_ = 6.607, p = 0.0107
- Interaction: F _(11, 248)_ = 10.99, p < 0.0001

| Bonferroni's multiple comparisons test | Predicted (LS) mean diff. | 95.00% CI of diff. | t (df:248) | Summary | Adjusted P Value |
| --- | --- | --- | --- | --- | --- |
| HMC Th13 | 3.218 | 0.2757 to 6.161 | 3.163 | * | 0.0211 |
| LMCv L1/L2 | -0.03900 | -2.982 to 2.904 | 0.038 | ns | >0.9999 |
| LMCd L4/L5 | 4.704 | 2.497 to 6.911 | 6.164 | **** | <0.0001 |
| LMCv L4/L5 | 4.028 | 1.748 to 6.307 | 5.110 | **** | <0.0001 |
| LMCd L6 | 4.145 | 0.5405 to 7.749 | 3.326 | * | 0.0122 |
| MMC Th13 | 0.8494 | -1.942 to 3.641 | 0.878 | ns | >0.9999 |
| MMC L1/L2 | -0.9560 | -4.077 to 2.165 | 0.886 | ns | >0.9999 |
| MMC L3/L4 | -0.1519 | -2.556 to 2.252 | 0.183 | ns | >0.9999 |
| MMC S1 | -4.833 | -7.152 to -2.513 | 6.025 | **** | <0.0001 |
| PGC Th13 | -0.4782 | -2.927 to 1.970 | 0.565 | ns | >0.9999 |
| PGC L1/L2 | -0.4767 | -3.268 to 2.315 | 0.494 | ns | >0.9999 |
| PGC S1 | -1.608 | -4.157 to 0.9403 | 1.825 | ns | 0.8305 |

***.***

**Supplementary file 1d;** related to Figure 5E, bottom graph.

**Foxp2-V1 vs CB+-V1 (Renshaw) synapses**

Two-Way ANOVA for Synapse origin and Motor Column/Segment

- Column/Segment: F_(11, 271)_ = 27.67, p < 0.0001
- Foxp2 vs CB+(Renshaw): F_(1, 271)_ = 77.60, p < 0.0001
- Interaction: F _(11, 248)_ = 14.71, p < 0.0001

| Bonferroni's multiple comparisons test | Predicted (LS) mean diff. | 95.00% CI of diff. | t (df:248) | Summary | Adjusted P Value |
| --- | --- | --- | --- | --- | --- |
| HMC Th13 | 4.348 | 1.971 to 6.725 | 5.286 | **** | <0.0001 |
| LMCv L1/L2 | 0.4498 | -1.927 to 2.827 | 0.547 | ns | >0.9999 |
| LMCd L4/L5 | 5.128 | 2.996 to 7.260 | 6.951 | **** | <0.0001 |
| LMCv L4/L5 | 6.682 | 4.751 to 8.613 | 9.999 | **** | <0.0001 |
| LMCd L6 | 5.632 | 3.078 to 8.187 | 6.371 | **** | <0.0001 |
| MMC Th13 | 0.8168 | -1.448 to 3.081 | 1.042 | ns | >0.9999 |
| MMC L1/L2 | -0.7661 | -3.110 to 1.578 | 0.945 | ns | >0.9999 |
| MMC L3/L4 | 2.165 | 0.1277 to 4.202 | 3.071 | * | 0.0282 |
| MMC S1 | -2.069 | -4.068 to -0.0705 | 2.992 | * | 0.0363 |
| PGC Th13 | 0.2166 | -1.732 to 2.165 | 0.321 | ns | >0.9999 |
| PGC L1/L2 | 0.5643 | -1.660 to 2.789 | 0.733 | ns | >0.9999 |
| PGC S1 | 0.03761 | -2.043 to 2.118 | 0.052 | ns | >0.9999 |

**Supplementary file 1e;** related to *Figure 6C (left: Otp/Foxp2).*

| Ordinary one-way ANOVA summary |  |
| --- | --- |
| F | 45.10 |
| P value | <0.0001 |
| P value summary | **** |
| Significant diff. among means (P < 0.05)? | Yes |
| R squared | 0.9442 |

| Bonferroni's multiple comparisons test | Mean Diff. | 95.00% CI of diff. | t(dg:8) | Summary | Adjusted P Value |
| --- | --- | --- | --- | --- | --- |
| None vs.  OTP and Foxp2 | -12.45 | -24.20 to -0.6918 | 3.684 | * | 0.0371 |
| None vs.  OTP only | 16.67 | 4.917 to 28.42 | 4.934 | ** | 0.0069 |
| None vs.  Foxp2 only | 22.87 | 11.11 to 34.62 | 6.768 | *** | 0.0009 |
| OTP and Foxp2 vs. OTP only | 29.12 | 17.36 to 40.87 | 8.618 | *** | 0.0002 |
| OTP and Foxp2 vs. Foxp2 only | 35.31 | 23.56 to 47.07 | 10.45 | **** | <0.0001 |
| OTP only vs. Foxp2 only | 6.195 | -5.559 to 17.95 | 1.834 | ns | 0.6243 |

**Supplementary file 1f;** related to *Figure 6C (center: Foxp4/Foxp2).*

| Ordinary one-way ANOVA summary |  |
| --- | --- |
| F | 81.53 |
| P value | <0.0001 |
| P value summary | **** |
| Significant diff. among means (P < 0.05)? | Yes |
| R squared | 0.9683 |

| Bonferroni's multiple comparisons test | Mean Diff. | 95.00% CI of diff. | t(dg:8) | Summary | Adjusted P Value |
| --- | --- | --- | --- | --- | --- |
| None vs.  Foxp4 and Foxp2 | -12.45 | 23.68 to 47.30 | 10.45 | **** | <0.0001 |
| None vs.  Foxp4 only | 16.67 | 39.97 to 63.59 | 15.25 | **** | <0.0001 |
| None vs.  Foxp2 only | 22.87 | 14.11 to 37.73 | 7.635 | *** | 0.0004 |
| Foxp4 and Foxp2 vs. OTP only | 29.12 | 4.478 to 28.10 | 4.798 | ** | 0.0082 |
| Foxp4 and Foxp2 vs. Foxp2 only | 35.31 | -21.38 to 2.241 | 2.819 | ns | 0.1352 |
| Foxp4 only vs. Foxp2 only | 6.195 | -37.67 to -14.05 | 7.617 | *** | 0.0004 |

**Supplementary file 1g;** related to *Figure 6C (right: Otp/Foxp4).*

| Ordinary one-way ANOVA summary |  |
| --- | --- |
| F | 117.3 |
| P value | <0.0001 |
| P value summary | **** |
| Significant diff. among means (P < 0.05)? | Yes |
| R squared | 0.9778 |

| Bonferroni's multiple comparisons test | Mean Diff. | 95.00% CI of diff. | t(dg:8) | Summary | Adjusted P Value |
| --- | --- | --- | --- | --- | --- |
| None vs.  OTP and Foxp4 | 26.05 | 17.11 to 35.00 | 10.14 | **** | <0.0001 |
| None vs.  OTP only | 21.27 | 12.32 to 30.21 | 8.273 | *** | 0.0002 |
| None vs.  Foxp4 only | 47.99 | 39.04 to 56.93 | 18.67 | **** | <0.0001 |
| OTP and Foxp4 vs. OTP only | -4.787 | -13.73 to 4.156 | 1.862 | ns | 0.5976 |
| OTP and Foxp4 vs. Foxp4 only | 21.93 | 12.99 to 30.88 | 8.533 | *** | 0.0002 |
| OTP only vs. Foxp4 only | 26.72 | 17.78 to 35.66 | 10.40 | **** | <0.0001 |
